# Subclinical Hypothyroidism: is it Really Subclinical with Aging?

**DOI:** 10.14336/AD.2018.0817

**Published:** 2019-06-01

**Authors:** Robin Gourmelon, Sandrine Donadio-Andréi, Karim Chikh, Muriel Rabilloud, Elisabetta Kuczewski, Anne-Sophie Gauchez, Anne Charrié, Pierre-Yves Brard, Raphaëlle Andréani, Jean-Cyril Bourre, Christine Waterlot, Domitille Guédel, Anne Mayer, Emmanuel Disse, Charles Thivolet, Hélène Du Boullay, Claire Falandry, Thomas Gilbert, Anne François-Joubert, Antoine Vignoles, Catherine Ronin, Marc Bonnefoy

**Affiliations:** ^1^Service de Gériatrie, Hospices Civils de Lyon, Lyon Sud Hospital, Pierre-Bénite, France.; ^2^Siamed’Xpress, Hôtel Technologique Morandat, 13120 Gardanne, France.; ^3^Hospices Civils de Lyon, 69002 Lyon, France.; ^4^Laboratoire de Biochimie et Biologie Moléculaire, Centre de Biologie Sud, Centre Hospitalier Lyon Sud 69 495 Pierre Bénite Cedex, France.; ^5^Société Française de Médecine Nucléaire, Groupe de Biologie Spécialisée, 75237 Paris Cedex 05, France.; ^6^Service de Biostatistiques, Hospices Civils de Lyon, Lyon, France.; ^7^UMR-S INSERM 1039, 38000 Grenoble, France.; ^8^Service de Médecine Nucléaire, Centre Hospitalier Métropole Savoie, 73000 Chambéry, France.; ^9^Pôle de Biologie, Centre Hospitalier et Universitaire de Grenoble, CS 10217 38043 Grenoble Cedex 9, France.; ^10^Service d’Endocrinologie, Centre Hospitalier Métropole Savoie, 73000 Chambéry, France

**Keywords:** thyroid deficiency, aging, symptoms, TSH

## Abstract

No recent study has focused on clinical features of subclinical hypothyroidism (SCH), especially in older patients. TSH measurement has remarkably evolved these last 20 years and thus reconsideration is needed. In our prospective multicenter study (2012-2014) including 807 subjects aged <60 years (<60y) and 531 subjects ≥60 years (≥60y), we have monitored 11 hypothyroidism-related clinical signs (hCS) together with TSH, FT4, FT3 and anti-thyroperoxidase antibodies values. hCS expression has been compared in patients with SCH *vs* euthyroidism in each age group. The number of hCS above 60y of age were found to be more elevated in the euthyroid population (1.9 *vs* 1.6, p<0.01) than in the SCH population (2.3 *vs* 2.6, p=0.41) while increase in hCS is limited to SCH subjects in the <60y group (p<0.01). The percentage of subjects with at least 3 signs increased with SCH in the <60y group (42.6% *vs* 25.0%, p<0.01) but not ≥60y (34.4% *vs* 33.9%, p=0.96). In older individuals, only three hCS could be related to both SCH and a decreased T3/T4-ratio (0.26 *vs* 0.27, p<0.01), suggesting either a reduced activity of TSH, or an adaptive response with aging. While hCS are clearly associated with SCH in patients <60y, they are not so informative in older subjects. TSH measurements carried out on the basis of hCS need to be interpreted with caution in aged patients. A reassessment of the TSH reference range in older patients is clearly needed and should be associated to more appropriate monitoring of thyroid dysfunction

Subclinical hypothyroidism (SCH) is defined by elevated TSH (Thyroid-Stimulating Hormone) values and normal levels of free thyroxin (FT4). Among thyroid diseases, this disorder remains elusive and may affect about 1-11% of the worldwide population (1-7). Such wide variability can be attributed to environmental/ethnic specificities, but it is worth noting that it may also originate in the TSH reference range used to define the presence of abnormal thyroid function which is still quite actively debated, especially among countries (8). The prevalence of SCH diagnosis increases with age and is the most common thyroid dysfunction in older subjects (9, 10, 11) reaching 14.5% in individuals ≥60 years (≥60y) (12) and up to 22% in women over 60 years (1). This disorder has long been particularly difficult to define as clinical signs may be present but not necessarily related to biological indicators. Virtually no progress has been made in the diagnosis of SCH over the recent past even though the pathology may be more deleterious with age (8). Recently, treatment with L-thyroxine in people above 65y with SCH failed to demonstrate efficacy (13), suggesting that the distribution of age groups according to hypothyroidism-related clinical signs (hCS) clinical signs and TSH level may not be adequate.

hCS are composed of a remarkable wide array, including signs of hypometabolism, cardiovascular, neuromuscular and mucocutaneous incidents. They have been often combined to establish clinical scores to help diagnosing thyroid deficiency: Wayne’s index (1959), Billewicz’s score (1969) or Zulewski’s score (1997) (Supplementary Table 1) (14). The latest score is based on ankle reflex, diminished sweating, hoarseness of voice, paraesthesia, dry, cold and coarse skin, constipation, impairment of hearing, weight increase, slow movements and periorbital puffiness (15). However, as all these symptoms may be altered by aging (8, 16), a cautious interpretation would be recommended for older patients (16, 17). Aging has been shown to alter the expression of clinical signs in overt hypothyroidism (17, 18). While recent studies have focused on more subjective quality of life questionnaires (19), the clinical symptomology of SCH has not been revised over the past 15 years and remains insufficiently described (1, 15) essentially because these signs often overlap with those of aging (20). Indeed, a single study has investigated the presence of hCS in older SCH patients (21) and supported the idea that a good correlation between hCS and TSH level is needed to improve early screening of thyroid deficiency. Definition of SCH according to TSH level has largely been reinvestigated as TSH testing has remarkably evolved through several generations of tests but the upper decision limit has not been yet settled, especially in the elderly. Epidemiological studies reported that the upper limit of the expected normal TSH increases with age (10, 11) and it remains unclear whether such elevation may reflect hypothyroidism or common healthy aging is actively debated (22).

In this study, we have explored the expression of 11 of the 12 hCS included in the Zulewski’score (15) in subjects <60y and ≥60y of age to putatively identify more relevant signs. hCS were estimated both as a whole panel and individually and further related to biological parameters.

**Figure 1.**
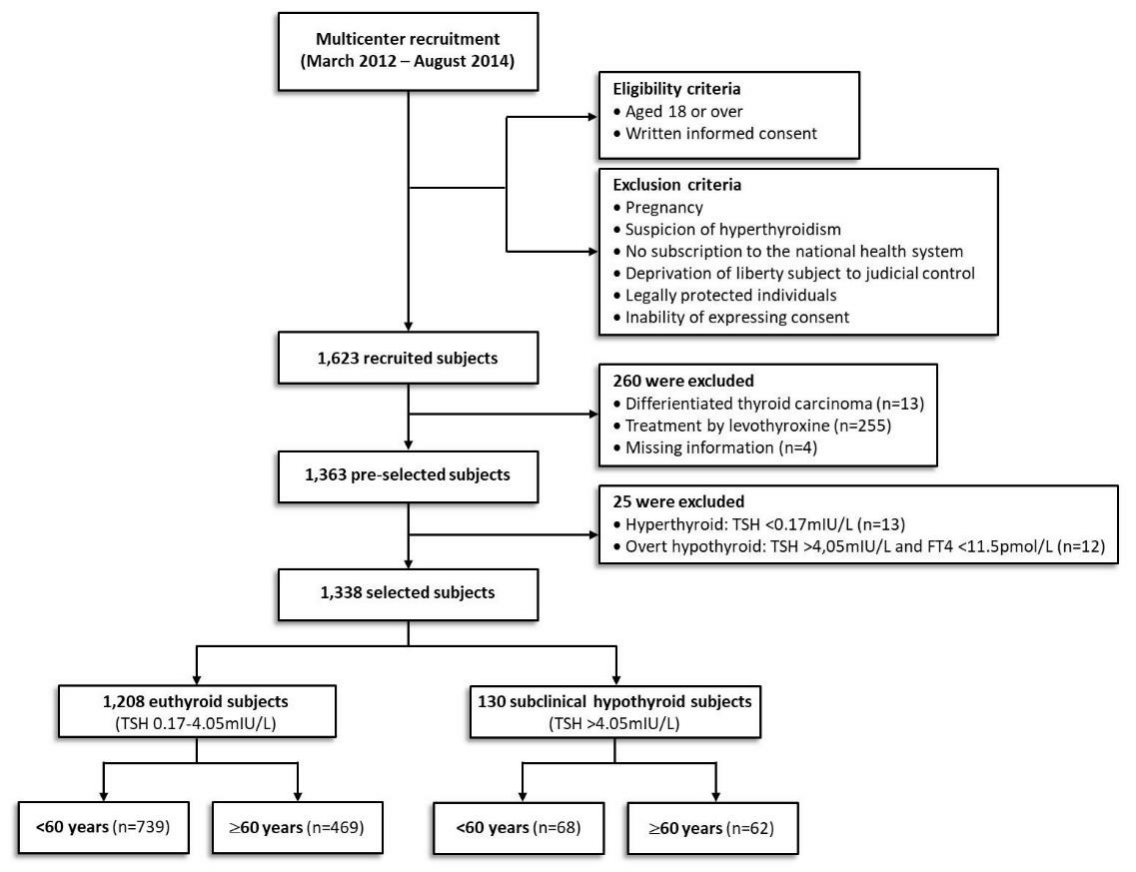
Flowchart of the recruitment, selection and exclusion process of the cohort.

## MATERIALS AND METHODS

### Study population

Recruitment of the prospective multicenter study was performed in the Departments of Endocrinology and Geriatrics at the Lyon-Sud Hospital (France), and in the Departments of Endocrinology and Nuclear Medicine at the Chambery Hospital (France). All men and women who consulted in these four hospital departments involved in the study were invited to participate. No announcement has been made. All patients for whom a TSH assay was prescribed by current care physicians were invited to participate, irrespective of any history of thyroid dysfunction, and gave informed consent. Patients with differentiated thyroid carcinoma, under thyroxine treatment or missing information were excluded. Hyperthyroid patients (TSH <0.17 mIU/L) were also excluded as well as patients with elevated TSH (>4.05 mIU/L) and low FT4 (<11.5 pmol/L) defined as overt hypothyroid. We used the upper limit of 4.05mIU/L for TSH as recommended by the assay manufacturer (IRMA, Beckman Coulter) and in accordance with the National “Haute Autorité de Santé” (HAS) standard set (23). The study was therefore based on 1,338 individuals (18-98 years) distributed in euthyroid (TSH, 0.17-4.05 mIU/L) and SCH (TSH>4.05 mIU/L FT4≥11.5 pmol/L) (Fig 1). These two groups were further investigated according to age *i.e.* <60y and ≥60y. The limit of 60 years was selected because earlier studies indicated that the 97.5^th^ centile increased beyond 4mIU/L (>4.2 mIU/L) after the age of 60 (24-26). Patients with TSH within the range of 4.1-10.0 mIU/L were also analysed according to the presence (>30 kU/L) or absence of anti-thyroperoxidase antibodies (TPOAbs), based on management practice as 80% of SCH patients fall in this range (27, 28). The study was approved by the Committee of Protection of the People, registered at ClinicalTrials.gov (NCT01997554) and cleared by the local Institution Ethics Review Board for human studies. Written consent forms for all patients were accepted by the HCL-DRCI. This work was supported by the French National Research Agency *via* the BIOTECS funding (grant number ANR-10-BIOT-0013)

### Serum assays and data collection

Samples were obtained at the time of inclusion (no follow-up). TSH was estimated (IRMA TSH, Villepinte, France) as well as FT4 (RIA, Beckman Coulter, Villepinte, France), FT3 (RIA, Beckman Coulter, Villepinte, France) and TPOAbs (RIA, ThermoFisher Scientific B.R. A.H.M.S, Asnières, France). Age, sex, thyroid-interfering medication (amiodarone, psychotropic and interferon) and the absence/presence of hCS as described by Zulewski and colleagues (13) have been recorded by clinicians to limit biases due to self-reporting by subjects. We did not include the ankle reflex for practical reasons and based our analysis on 11 hCS. All data were entered in Case Report Forms by the Clinical Research Assistant and the elaboration of the database was outsourced to ClinInfo (Lyon, France).

### Data analysis

The diagnostic performance of each hCS was quantified using sensitivities, specificities, likelihood ratios (LR) within a 95% confidence interval (CI). Logistic regression models were used for quantifying the effect of the hCS and predicting the probability of SCH according to the combinations of signs. Empirical ROC curves were built using predicted probabilities. Diagnostic performance was quantified by estimation of the area under the ROC curves (AUROCs) with its 95% CI. AUROCs were compared using the non-parametric method of Delong. Comparisons of means and of percentages were carried out using respectively ANOVA and chi-square test. Analyses were carried out using the statistical software SPSS version 21 and R version 3.2.1. For all the comparisons a p<0.05 was considered for the statistical significance.

**Table 1:** Mean number of overall clinical signs.

Groups & Classes	<60 years		≥60 years	
Euthyroid**	1.6±1.6	(n=739) ††	1.9±1.7	(n=469)
SCH	2.6±2.1	(n=68)	2.3±2.3	(n=62)

*4.1-10.0mIU/L range:*				
TPOAbs (-)	2.4±1.9	(n=37)	1.8±2.0	(n=42)
TPOAbs (+)	3.0±2.3	(n=28)	2.7±2.3	(n=16)

Results are presented as mean±SD. Groups overwritten with an asterisk are significantly different between <60y and ≥60y (**, p<0.01). Values overwritten with crosses are significantly different between TSH groups, *i.e.* between euthyroid (0.17-4.05mIU/L) and subclinical hypothyroid (SCH, >4.05mIU/L) groups or between the absence/presence of TPOAbs when TSH is in the 4.1-10.0mIU/L range (††, p<0.01).

**Figure 2.**
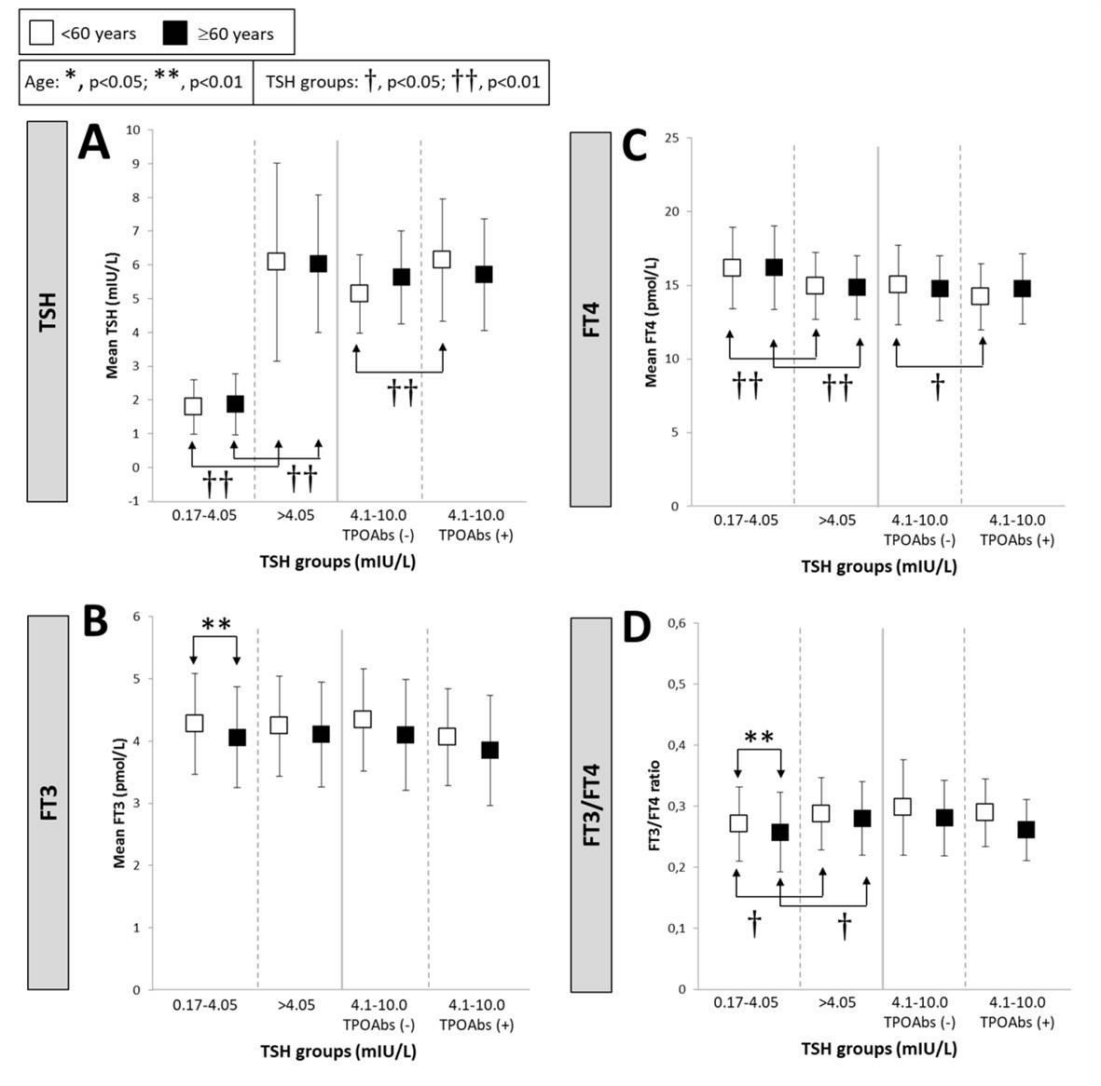
Mean TSH (A), FT3 (B), FT4 (C) levels and FT3/FT4 ratios (D) as a function of TSH levels Asterisks indicate significant differences between age groups (**, p<0.01) and crosses indicate significant differences between normal (0.17-4.05 mIU/L) and elevated (>4.05 mIU/L) TSH groups or difference between the absence/presence of TPOAbs when TSH is in the 4.1-10.0mIU/L range (†, p<0.05; ††, p<0.01).

## RESULTS

### Description of the population and prevalence of SCH

The average age of the cohort was of 43±11 years for the <60y group and of 71±9 years for the ≥60y population. Females were more represented in the younger participants. 12.9% of the subjects were under medication in the <60y group and 16.0% in the ≥60y (p=0.13) (Supplementary Table 2) and the inclusion of such patients in the study did not modify the results of the study (data not shown). TSH levels were higher (p<0.05) and FT3 levels lower (p<0.01) in older individuals.

The proportion of SCH was 9.7% (Supplementary Table 3). A higher incidence was observed in the ≥60y (11.7%) compared to <60y individuals (8.4%) (p=0.06). SCH subjects were more likely to have TPOAbs than euthyroid individuals (36.9% *vs* 14.7%, p<0.01). Auto-immunity was similar in both age groups (3.0% vs 3.5% for ≥60y and <60y respectively, p=0.10). Furthermore, the percentage of subjects without TPOAbs and with a TSH betwin 4.1-10.0 mIU/L was higher in seniors (7.9% vs 4.6%, p<0.05).

**Figure 3.**
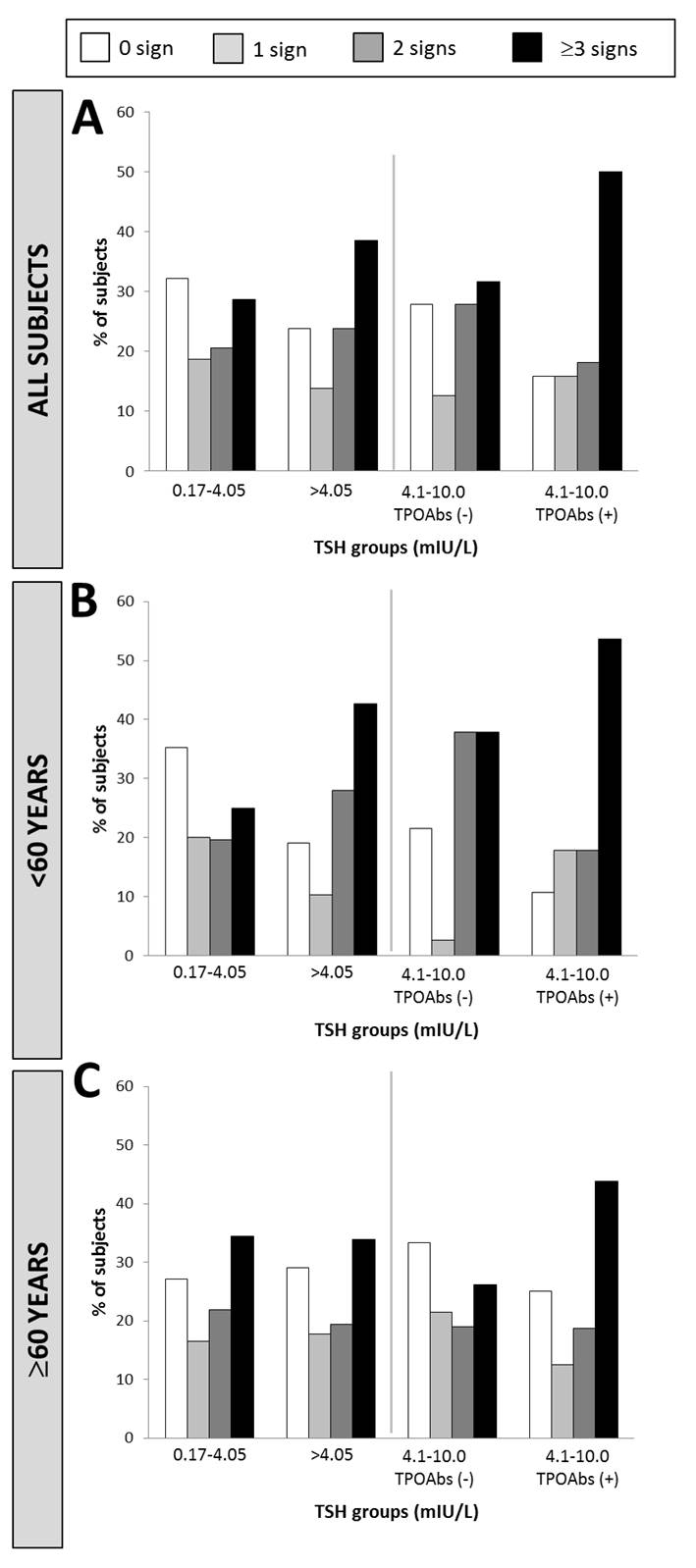
Percentage of subjects with 0, 1, 2, or 3 or more clinical signs by TSH groups Data from all subjects (**A**), subjects <60y (**B**) and ≥60y (**C**) are presented.

**Figure 4.**
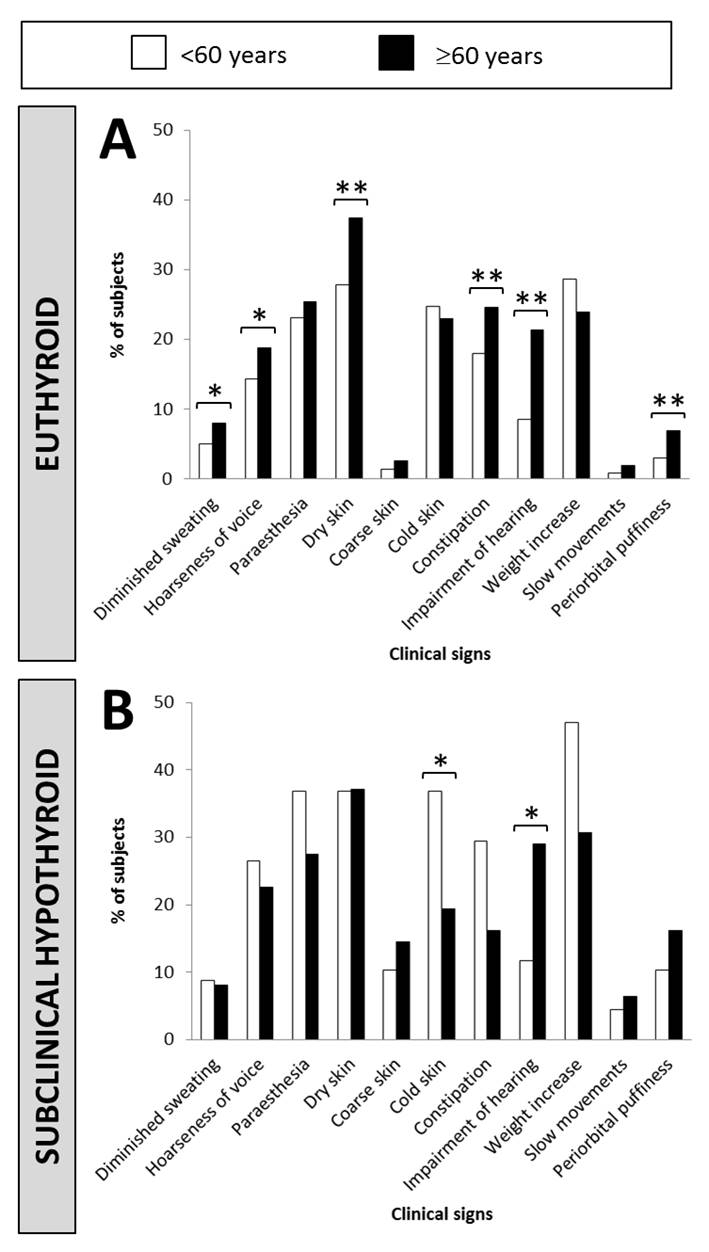
Prevalence of individual hypothyroidism-related clinical signs according to age Age groups are <60y (white bars) and ≥60y (black bars). A) euthyroid subjects (TSH, 0.17-4.05mIU/L); B) subclinical hypothyroid subjects (TSH, >4.05mIU/L). Bars with an asterisk are significantly different between <60y and ≥60y groups (*p<0.05; **p<0.01).

### Biological parameters

The mean TSH level was significantly higher in ≥60y (2.35±1.73) than in the younger group (2.15±1.66) (p<0.05). For <60y individuals in the range of 4.1-10.0mIU/L, TSH was higher in the presence of autoimmunity (Fig. 2A). FT3 values were reduced only in euthyroid older persons (Fig. 2B). FT4 levels were similar between age groups but significantly decreased when TSH increased (Fig. 2C). As a consequence, the FT3/FT4 ratio decreased in euthyroid older subjects with normal TSH and increased with elevated TSH values in both age groups (Fig. 2D).

### Overall expression of hCS

The number of hCS was significantly higher in patients with higher TSH levels (p<0.01). However, this was true only for the population of younger but not for older subjects (table 1). No difference was observed with regards to FT4 levels in either age groups (p>0.71) (data not shown). In patients with SCH, no difference was found between age groups. Among euthyroid patients, hCS were significantly more frequent in older compared to younger subjects (p<0.01) (table 1).

Figure 3 pictures graphically that patients with no hCS represented the largest proportion individuals in the euthyroid group, while patients with three or more hCS were more frequent in the SCH group (p<0.05) (Fig. 3A, left side). However, when considering patients aged ≥60 years, the number of hCS did not differ significantly with TSH levels (Fig. 3C, left side). In both age groups, hCS seemed more frequent in presence of anti-TPO antibodies (Table 1 and Fig. 3, right side). However, this finding was not statistically significant.

Also, estimating the AUROCs showed no impact of age on the increase of hSC (p=0.28): 68% (CI 95%, 0.61-0.75) and 62.4% (CI 95%, 0.55-0.70) for <60y and ≥60y groups respectively (Supplementary Fig. 1) as well as between males and females (Supplementary Fig. 2).

### Expression of individual hCS

In the <60y subjects, weight increase (OR 1.75; CI 95%, 1.02-3.03, p=0.04) and coarse skin (OR 6.64; CI 95%, 1.93-22.81, p=0.003) were associated with SCH while in older subjects, SCH was associated with periorbital puffiness (OR 2.42; CI 95%, 1.01-5.84) and also coarse skin (OR 7.3; CI 95%, 2.48-21.85) (p<0.001). Of interest, the younger group displayed up to 4 hCS with high specificity (≥95%) (97%, periorbital puffiness; 99%, slow movements; 99%, coarse skin; 95%, diminished sweating) whereas only 2 of them could be highlighted in the ≥60y (98%, slow movements; 97%, coarse skin). Sensitivities were low for both groups (<50%) (Supplementary Table 4). Height hCS had good positive LR in the <60y group but only 3 in the >60y group. Three hCS showed LR >2 in both age groups: periorbital puffiness, slow movement and coarse skin. Most of negative LR was near the value of 1. Only weight increase, coarse skin and paraesthesia in subjects <60y, plus coarse skin in older individuals were found significant (Supplementary Table 5).

We also observed that older subjects presented more frequently diminished sweating and hoarseness of voice (p<0.05), dry skin, constipation, impairment of hearing and periorbital puffiness (p<0.01) in the euthyroid population (Fig. 4A), while only impaired hearing was more represented (p<0.05) in the SCH population. Cold skin was also less frequent in the older compared to younger subjects (p<0.05) (Fig. 4B). Regarding TSH level, the prevalence of 8 hCS was increased in younger SCH subjects compared to euthyroid individuals: paraesthesia, cold skin, constipation (p<0.05), hoarseness of voice, coarse skin, weight increase, slow movements and periorbital puffiness (p<0.01) (Supplementary Fig. 3). In contrast, in older individuals, only 3 hCS were more frequent when TSH was in the subclinical range (p<0.05, slow movements and periorbital puffiness; p<0.01, coarse skin). In these subjects, we also observed that weight increase was less frequent in the absence of autoimmunity (p<0.05) and that constipation (p<0.05) and periorbital puffiness (p<0.01) were more frequent with autoimmunity. Slow movements were more frequent in younger SCH subjects with TPOAbs (p<0.05) (data not shown).

In addition, to determine which signs could be determined as nonspecific symptoms, we performed an F-test based on normal (NACB recommendations) and hypothyroid populations of our cohort. We found that 4 signs showed low Relative Risk Values: paraesthesia (1.42), constipation (1.36), dry skin (1.23) and cold skin (1.18). If we base our analysis on the 7 remaining clinical signs, main results were not affected (data not shown).

Finally, we found about 10% more females than males in the <60 group compared to the ≥60y group. To control this point, we compared the expression of clinical signs between males and females and found that it does not modify our conclusion, but probably minimize it.

## DISCUSSION

In this study, we showed that clinical features of SCH decreased with age *i.e.* ≥60y and observed no satisfactory correlation to usual biological parameters of thyroid testing in the elderly.

### Expression of clinical signs

Six hCS were more represented in older than in younger euthyroid subjects (p<0.01): periorbital puffiness, impairment of hearing, constipation, dry skin and diminished sweating and hoarseness of voice but these signs are also described in physiological aging (29-33). In SCH seniors, coarse skin and slow movements showed increased expression, correlated with high specificity and good positive LR. Coarse skin may reflect mucocutaneous infiltration and hypometabolism which may evolve into myxoedematous conditions (34) and slow movements have been described as early signs of hypothyroidism (35). However, the poor sensitivity (<15%) and positive LR, as observed in the COLORADO study (1) prevent these signs for being truly reliable for SCH screening.

Earlier studies proposed combined evaluation of hCS (15, 17, 21) but only Bemben et al. in 1994 (21) distinguished SCH from overt hypothyroidism in older persons based on the recruitment of 284 subjects (65-97 years) including 42 SCH patients. Recruiting a larger cohort (531 subjects aged 60-98 years, including 62 SCH patients) allowed us to show that the predictive potency for the whole hCS pattern did not differ so much with age. Similar findings were previously reported without distinction between SCH and hypothyroidism (1). We could not find a strong expression of hCS in SCH patients over 60y in spite of a positive relationship between the number of hCS and TSH levels. Also, the percentage of subjects with 3 or more hCS was not significantly higher in older SCH individuals compared to euthyroid subjects, except in the presence of TPOAbs. There may therefore be a distinct clinical expression due to the presence of anti-TPO antibodies, which would not be predicted by the elevation of TSH. Given that some of our results were not significant, we can not conclude on this supposition, but this can be a fruitful search track to consider. By the way, we did not observe a reduced number of signs as reported in older hypothyroid patients (17, 18). We thus concluded that hCS should be carefully investigated with aging as they are moderately specific and that only some of them may correlate with biological parameters.

### Biological parameters

The SCH prevalence of 9.7% is in good agreement with the 9.2-9.5% reported so far (1, 6). As previously published (10, 11), the mean TSH level increased with aging to reach a prevalence of a diagnosed SCH prevalence of 11.7% (8.4% for <60y group), in agreement with the 10.4-14.5% found in European citizens ≥60y (12, 36). Unfortunately, given the context of the study, which was conducted in current care, we were not able to perform follow up measurements of thyroid function markers or to follow evolution of the diagnosed SCH prevalence in our cohort. This could however be a trail for future studies.

A similar proportion of the <60y and ≥60y participants were under medication, suggesting that elevated TSH was not due to polypharmacy in the elderly (24). A physiological increase in TSH level has been consistently reported with aging (11, 12, 24, 37, 38). Alteration of the hypothalamic-pituitary axis is supported by a change in the pituitary threshold for the TSH feedback suppression, loss of TSH nocturnal peak and decreased thyroid hormone levels (39), as well as damage of thyroid cells by reactive oxygen species (40). Elevated TSH level which is not on line with T3 and T4 levels together with hCS could be indicative of reduced bioactivity of TSH (22, 25, 41). Such change may explain the discrepancy observed between TSH level and a poor clinical expression. Whether it is a normal adaptive response to aging or a progressive alteration of the thyrotroph axis is still unclear (8). In euthyroidism, reduced FT3 levels may occur upon aging independently of FT4 level, leading to a decreased FT3/FT4 ratio in older subjects and reduced stimulation of the thyroid (25, 42): when TSH bioactivity declines, a greater concentration of the hormone is required to stimulate the thyroid and maintain a physiological level of T4 in the circulation (25). Finally, both age groups exhibited ’3% of subjects with TPOAbs. The presence of TPOAbs appeared associated to increased TSH and reduced FT4 levels in <60y individuals, but not in seniors, suggesting that TPOAbs correlate with SCH in <60y subjects (2) but not in older participants (43) for whom it may rather indicate immune senescence (44). Moreover, while TPO antibodies are clearly associated with increased risk of hypothyroidism, this finding could corroborate physiologically increased levels of TSH with aging.

### Detection of SCH with aging

Our results clearly indicate that the clinical evaluation of SCH is insufficiently reliable for patients aged 60 years or older. The clinical evidence of thyroid dysfunction is barely detectable and only abnormality is a marginally elevated TSH. As a result, the SCH diagnosis should be made with caution in older people. Accordingly, treatment with levothyroxine in older persons with SCH may fail to provide no symptomatic benefits (13). To better detect SCH in the elderly, several approaches should be envisaged. Firstly, the elaboration of an age-related TSH reference range (11, 24, 37) may avoid misclassification of patients, suboptimal management and possible iatrogenic risks (11, 12). Secondly, assays measuring the level of bioactive TSH using newly designed assays (45) may better relate TSH to both hormonal activity (22) and clinical signs (46).

### Conclusions

Among the 11 evaluated hCS, periorbital puffiness, slow movements and mostly coarse skin appeared rather specific for SCH in older patients. However, the low sensitivity of hCS and the altered levels of TSH with aging make the diagnosis of SCH difficult in older individuals. Defining an age-related reference range and/or developing assays able to measure bioactive TSH is needed to improve SCH detection in the elderly.
